# Replacing of sedentary behavior with physical activity and the risk of mortality in people with prediabetes and diabetes: a prospective cohort study

**DOI:** 10.1186/s12966-023-01488-0

**Published:** 2023-07-06

**Authors:** Ping Zhu, Guojuan Lao, Huipeng Li, Rongshao Tan, Jing Gu, Jianmin Ran

**Affiliations:** 1 Department of Endocrinology and Metabolism, Guangzhou Red Cross Hospital, Jinan University, Guangzhou, China; 2 Institute of Disease-Oriented Nutritional Research, Guangzhou Red Cross Hospital, Jinan University, Guangzhou, China; 3 Department of Endocrinology and Metabolism, Sun Yat-Sen Memorial Hospital, Sun Yat-Sen University, Guangzhou, China; 4 Department of Medical Statistics, School of Public Health, Sun Yat-Sen University, Guangzhou, China

**Keywords:** Sedentary behavior, Mortality, Isotemporal substitution model, Diabetes, Prediabetes

## Abstract

**Background:**

Sedentary behavior is prevalent among people with diabetes and is associated with unfavorable cardiometabolic health. However, there is limited evidence regarding the impact of replacing sedentary time (ST) with physical activity on mortality in people with prediabetes and diabetes. We prospectively examined the association between accelerometer-measured ST and mortality among people with prediabetes and diabetes after adjusting for demographic characteristics, lifestyle factors, and moderate- to vigorous-intensity PA (MVPA). We further determined the effect of replacing ST with equal time of different types of physical activities on all-cause mortality.

**Methods:**

We included 1242 adults with prediabetes and 1037 with diabetes from the National Health and Nutrition Examination Survey. Restricted cubic splines were fitted to determine the dose–response association between ST and overall mortality. Isotemporal substitution modeling was used to explore the hazard ratio (HR) effects of ST replacement.

**Results:**

During a median follow-up of 14.1 years, 424 adults with prediabetes and 493 with diabetes died. Compared with the lowest tertile of ST, the multivariable-adjusted HRs for all-cause mortality in the highest tertile were 1.76 (95% confidence interval [CI] 1.19, 2.60) for participants with prediabetes and 1.76 (1.17, 2.65) for those with diabetes. Additionally, a linear association between ST and all-cause mortality was observed in adults with prediabetes and diabetes, with HRs for each 60 min/day increment in ST of 1.19 (1.10, 1.30) and 1.25 (1.12, 1.40), respectively. Isotemporal substitution results indicated that individuals with prediabetes whose ST was replaced by 30 min of light-intensity physical activity (LPA) and MVPA had 9% and 40% lower all-cause mortality, respectively. In people with diabetes, replacing sedentary behavior with an equivalent time of LPA and MVPA was also associated with mortality risk reduction (HR 0.89; 95% CI 0.84, 0.95 for LPA; HR 0.73; 95% CI 0.49, 1.11 for MVPA).

**Conclusions:**

Higher ST was associated in a dose–response manner with an increased risk of premature mortality among adults with prediabetes and diabetes. Statistically replacing ST with LPA was potentially beneficial for health in this high-risk population.

**Supplementary Information:**

The online version contains supplementary material available at 10.1186/s12966-023-01488-0.

## Background

Diabetes affected more than 536 million people worldwide in 2021, and its prevalence is increasing [1, 2]. Prediabetes is associated with a greater risk of progression to diabetes. The global prevalence of impaired glucose tolerance (IGT) and impaired fasting glucose (IFG) in 2021 was 9.1% (464 million) and 5.8% (298 million), respectively, among adults aged 20–79 years [3]. The rising morbidity and mortality of prediabetes and diabetes underscore the need to identify additional modifiable risk factors such as physical inactivity and poor diet [4]. Over the past decades, sedentary behavior, defined as any waking activity with low energy expenditure while in a sitting, reclining, or lying position, has been increasing significantly in adults [5, 6]. Epidemiological evidence has indicated that sedentary duration is associated with all-cause and cardiovascular mortality in general population [7–9]. Therefore, recent national and global activity guidelines for adults have recommended limiting sedentary behavior in addition to physical activity [5, 10].

Adults with diabetes are more likely to engage in prolonged sedentary behavior than those without diabetes, largely due to barriers related to their symptoms, such as fatigue and intolerance to exercise [11, 12]. Sedentary behavior is associated with endothelial dysfunction and low-grade inflammation biomarkers in individuals with prediabetes and diabetes [11]. Additionally, increased sedentary time in patients with diabetes is also associated with a higher prevalence of carotid plaque and incident cardiovascular disease (CVD) [13]. The American Diabetes Association also recommended that adults with diabetes should decrease the amount of time spent in daily sedentary behavior [14]. Nevertheless, evidence regarding sedentary behavior and mortality risk in this specific subpopulation with prediabetes and diabetes is scarce and limited to self-reported sedentary measures or short follow-up periods, which may be subject to measurement errors and reporting bias [15–17].

Isotemporal substitution analysis, a widely used time-use statistical method in physical activity epidemiologic research, has been developed and applied to investigate the effect of replacing time spent in one behavior with another on health outcomes [18–21]. A prospective cohort study conducted on the general population, with a follow-up period of 15 years, found that substituting time spent engaged in light-intensity physical activity (LPA) or moderate- to vigorous-intensity PA (MVPA) with sedentary time increased the risk of all-cause mortality [20]. Emerging studies have indicated that the decreased risk of diabetes and all-cause mortality might be attributed to the replacement of sedentary behavior with physical activity in the general population [19, 22, 23]. In a recent study of adults with prediabetes and overweight/obesity from eight countries, Swindell et al. reported that replacing sedentary behavior with LPA or MVPA was associated with improved cardiometabolic risk markers [24]. However, the extent to which the substitution of sedentary behavior for physical activity reduces the risk of mortality among individuals with prediabetes and diabetes is largely unknown. Further understanding of the displacement effect of sedentary behavior with varying levels of physical activity on clinical outcomes would provide evidence for the development of targeted behavioral interventions in this vulnerable population.

The current study aimed, for the first time, to prospectively investigate the dose–response relationships between accelerometer-measured sedentary behavior and all-cause mortality risk in middle-aged and older adults with prediabetes and diabetes. To determine which kind of physical activity should be substituted for sedentary behavior to achieve health benefits, we also explored the effects of reallocating time spent in sedentary behaviors to LPA and MVPA on all-cause mortality using isotemporal substitution analyses.

## Methods

### Study population

The National Health and Nutrition Examination Survey (NHANES) is a nationally representative study designed to characterize the health and nutrition condition of the US population. The integration of the NHANES with the National Death Index database is commonly used as a prospective cohort study. The data collection and sampling methods for the survey are described elsewhere [25]. Both the National Center for Health Statistics and the Ethics Review Board approved the NHANES protocol (#98–12 and #2005–06). Written informed consent was obtained from each subject.

Our study involved two cycles of NHANES data from 2003 to 2006. The analysis included individuals aged 40 or more years with prediabetes and diabetes. Diabetes was identified based on a self-reported physician diagnosis of diabetes and elevated levels of fasting glucose (≥ 7.0 mmol/L), 2-h glucose during oral glucose tolerance test (≥ 11.1 mmol/L), or HbA1c (≥ 6.5%). Prediabetes was defined as an individual without diabetes but meeting one or more of the following criteria: self-reported physician diagnosis of prediabetes, fasting glucose concentration of 5.6–6.9 mmol/L, 2-h glucose during oral glucose tolerance test of 7.8–11.0 mmol/L, or HbA1c levels of 5.7–6.4%.

A total of 1600 adults with prediabetes and 1321 with diabetes were recruited. In the principal analysis, 1242 participants with prediabetes and 1037 participants with diabetes were included after excluding those who self-reported pregnancy (*n* = 35), lacked valid accelerometry data (*n* = 604), or were lost to follow-up (*n* = 3) (Supplementary Fig. 1). This study followed the Strengthening the Reporting of Observational Studies in Epidemiology (STROBE) reporting guideline.

### Sedentary behavior and physical activity

In this study, we measured sedentary behavior and physical activity using an AM-7164 accelerometer (ActiGraph, Pensacola, FL, USA). The methods used for collecting and managing accelerometer data are described in detail elsewhere [26]. In brief, eligible participants with no impairments preventing them from walking or wearing accelerometers were recruited during a physical examination at the mobile examination center. The accelerometer was worn on the hip for 7 consecutive days during waking hours, except when swimming or showering. Activity counts were recorded by the accelerometer in 1-min epochs, reflecting the intensity of body movement. At least 60 min of zero counts were required for non-wearing time, with no more than 2 min of counts between 1 and 100 allowed. An analysis was conducted on participants who recorded wear times of ≥ 10 h per day for at least 2 consecutive days. The daily time spent at different intensity levels was determined based on previously established cutoff methods [27, 28]. Each behavior was categorized using intensity threshold values of counts per minute (CPM) for adults: < 100 for sedentary behavior, 100–2020 for LPA, and ≥ 2020 for MVPA. Using the valid days that the accelerometer was worn, we estimated the means for each activity intensity category.

### Ascertainment of mortality

The National Death Index, linked with NHANES, was used to determine all-cause and CVD mortality through December 31, 2019. We defined the follow-up period as the time between the baseline measure of physical activity and either study outcomes or the end of follow-up, whichever happened first. ICD-10 codes I00–09, I11, I13, I20–51, and I60–69 represented deaths from CVD.

### Assessment of covariates

Baseline data from household interviews on age, sex, race/ethnicity, education level, family economic status, smoking status, alcohol consumption, and illness using standardized questionnaires were collected. In addition, data on body weight and height were obtained when participants were examined at a mobile examination facility. Body mass index (BMI) was determined as weight in kilograms divided by the square of height in meters and then categorized into three groups. Diet quality was assessed using healthy eating index-2015 (HEI) scores, with a higher score suggesting a healthier diet. Mobility limitations were defined as an inability to walk a quarter mile or up 10 stairs. The duration of diabetes was calculated according to the number of years between diabetes onset and baseline assessment for patients who had been diagnosed with diabetes and 0 years for patients who had not yet been diagnosed. The samples were divided into low- and high-activity subgroups based on the median of total LPA and MVPA times [23, 26]. Cardiometabolic biomarkers were also measured at the baseline survey, and detailed instructions for blood collection were provided in the NHANES Laboratory Manual.

### Statistical analyses

We utilized sample weights, pseudo-stratum, and primary sampling units to address the multistage sampling methodology used in the NHANES. Categorical variables are presented as n (weighted %) and continuous variables are presented as the weighted median (interquartile range). We compared the distribution of baseline characteristics between individuals with prediabetes and those with diabetes using the weighted χ2 test and Kruskal–Wallis test, respectively.

The survival status of the participants across tertiles of sedentary time was determined by weighted Kaplan–Meier curves. Sedentary behavior (by tertiles) and mortality risk from all-cause and CVD were investigated with survey-weighted Cox proportional hazards models. The median value of each category was used as a continuous variable to examine the linear trends. We developed three multivariable-adjusted models to account for the possibility of confounding variables. Model 1 was adjusted for age (10-year intervals), sex (female or male), race/ethnicity (non-Hispanic white, non-Hispanic black, Hispanic Mexican, or other), and total wear time (continuous). Model 2 was additionally modified for education level (less than high school, high school or equivalent, or college or above), family income-to-poverty ratio (< 1.0, 1.0–3.0, or ≥ 3.0), smoking status (never, former, or current smoker), alcohol consumption (yes or no), total cholesterol (TC) (continuous), high-density lipoprotein cholesterol (HDL-C) (continuous), glycated hemoglobin A1c (HbA1c) (continuous), and prevalence of CVD, hypertension, or cancer (yes or no). Diabetes duration (< 5 or ≥ 5 years) and glucose-lowering medications (insulin, oral antidiabetic agents only, or neither) were specifically adjusted for patients with diabetes. Model 3 was additionally modified for BMI (< 25.0, 25.0–29.9, or ≥ 30.0 kg/m^2^) and MVPA (continuous). The missing data were coded as a separate category for variables with a missing rate greater than 5%; otherwise, missing values were imputed as medians for continuous variables or modes for categorical variables [29].

The Cox proportional hazards models were then repeated with full adjustment and stratification by age (< 65 or ≥ 65 years), sex (female or male), race/ethnicity (non-Hispanic white or others), smoking (never or ever), alcohol consumption (never or ever), BMI (< 30 or ≥ 30 kg/m^2^), total physical activity level (low: < 5.6 h/day or high: ≥ 5.6 h/day for prediabetes; and < 4.9 or ≥ 4.9 h/day for diabetes; median split), and HbA1c level (< 7 or ≥ 7% for diabetes). A likelihood ratio test was used to identify the interactions between stratifying variables and sedentary time by comparing models with and without cross-product terms. A restricted cubic spline model with four knots (5th, 35th, 65th, and 95th) was used to examine the continuous dose–response relationship between sedentary time and mortality in people with prediabetes and diabetes, with the multivariate adjustment described above [30].

To determine whether physical activity at various intensities was a healthier alternative to sedentary behavior, we conducted isotemporal substitution analyses to investigate the effect of replacing sedentary time with equivalent amounts of LPA and MVPA on mortality risks [21]. The isotemporal substitution analyses make a more realistic assumption that an increase in one behavior will be accompanied by a decrease in the equivalent duration (isotemporal) of another behavior while the total time for all behaviors is fixed. Thus, a basic proportional hazards regression model was developed to measure the effect on all-cause mortality of replacing 30 min of sedentary behavior with LPA. The model incorporated LPA, MVPA, total wear time, and other covariates but omitted sedentary time. The resulting coefficients represented the consequence of reallocating time spent in sedentary behaviors to LPA or MVPA [31].

In sensitivity analyses, we excluded deaths within the first 2 years of follow-up to reduce the possibility of reverse causality. We reanalyzed the data after excluding those with a prevalence of CVD or cancer at baseline. We also reanalyzed the data after combining participants with prediabetes and diabetes. The multivariate model was additionally adjusted for mobility-limiting status and HEI-2015, which may influence the association of interest. Finally, to address whether the potential mediator, systemic inflammation, may have contributed to the observed association, C-reactive protein (CRP) was further adjusted for in a subgroup of the study individuals [32].

Statistical analyses were conducted using R software version 4.1.2. All statistical tests were two-sided, with *P* ≤ 0.05 indicating statistical significance.

## Results

### Baseline characteristics

The baseline characteristics of the study participants with weighted estimates according to tertiles of sedentary time are shown in Table 1. We included 1242 participants with prediabetes (median age, 56.0 years; 45.6% female) and 1037 participants with diabetes (median age, 62.0 years; 49.7% female). Overall, people with greater sedentary time were more likely to be older and non-Hispanic white; have higher BMI and education levels; have prevalent hypertension and CVD; and show lower HDL-C. Participants with prediabetes tended to be non-Hispanic white, have lower sedentary time, and have higher LPA and MVPA. Conversely, participants with diabetes tended to be older, have lower education levels and family income, and have higher BMI and prevalent hypertension and CVD.

**Table 1:** Characteristics of people with prediabetes and diabetes by tertiles of sedentary time

Characteristic	Sedentary behavior, h/day								P*
	**Prediabetes**				**Diabetes**				
	**Tertile 1** **(*N* = 414)**	**Tertile 2** **(*N* = 414)**	**Tertile 3** **(*N* = 414)**	**Total** **(*N* = 1242)**	**Tertile 1** **(*N* = 346)**	**Tertile 2** **(*N* = 345)**	**Tertile 3** **(*N* = 346)**	**Total** **(*N* = 1037)**	
Age, years	53.0 (47.0, 61.0)	58.0 (50.0, 69.0)	62.0 (51.0, 73.0)	56.0 (49.0, 68.0)	58.0 (50.0, 66.0)	62.0 (53.0, 71.0)	65.0 (56.0, 74.0)	62.0 (53.0, 71.0)	**< 0.001**
Female	213 (49.3)	193 (50.1)	160 (41.7)	566 (45.6)	173 (51.1)	180 (54.6)	162 (48.5)	515 (49.7)	0.094
Race/ethnicity									**0.010**
Non-Hispanic white	188 (70.0)	242 (78.5)	241 (76.2)	671 (54.0)	109 (59.5)	158 (71.7)	175 (71.2)	442 (42.6)	
Non-Hispanic black	92 (11.8)	88 (10.8)	91 (11.3)	271 (21.8)	75 (14.6)	89 (13.8)	104 (16.6)	268 (25.8)	
Hispanic Mexican	104 (8.8)	63 (3.4)	52 (3.5)	219 (17.6)	142 (15.3)	77 (5.4)	49 (3.9)	268 (25.8)	
Others	30 (9.4)	21 (7.3)	30 (9.0)	81 (6.5)	20 (10.6)	21 (9.1)	18 (8.3)	59 (5.7)	
Family income-poverty ratio									**< 0.001**
<1.0	62 (8.5)	46 (7.0)	56 (8.3)	164 (13.2)	84 (14.1)	66 (11.7)	50 (9.7)	200 (19.3)	
1.0–3.0	210 (45.6)	186 (35.3)	186 (38.2)	582 (46.9)	165 (46.8)	179 (49.3)	173 (44.6)	517 (49.9)	
≥ 3.0	142 (45.9)	182 (57.7)	172 (53.5)	496 (39.9)	97 (39.0)	100 (39.0)	123 (45.6)	320 (30.9)	
Education level									**0.011**
Less than high school	92 (11.6)	63 (8.5)	67 (8.6)	222 (17.9)	113 (16.7)	68 (11.6)	65 (11.7)	246 (23.7)	
High school or equivalent	182 (44.9)	153 (33.5)	152 (33.2)	487 (39.2)	131 (40.4)	157 (44.5)	130 (37.5)	418 (40.3)	
College or above	140 (43.4)	198 (58.0)	195 (58.1)	533 (42.9)	102 (42.9)	120 (43.9)	151 (50.8)	373 (36.0)	
Smoking status									0.527
Never	198 (45.8)	179 (44.3)	187 (45.2)	564 (45.4)	162 (47.6)	158 (50.4)	158 (46.1)	478 (46.1)	
Former	127 (30.1)	170 (38.7)	158 (39.1)	455 (36.6)	114 (33.0)	134(32.7)	133 (36.6)	381 (36.7)	
Current	89 (24.1)	65 (17.0)	69 (15.7)	223 (18.0)	70 (19.4)	53 (16.9)	55 (17.2)	178 (17.2)	
Nondrinker	127 (27.7)	121 (27.7)	141 (33.1)	389 (31.3)	128 (36.6)	144 (41.2)	124 (34.1)	396 (38.2)	**0.018**
BMI, kg/m^2^	29.6 (25.9, 34.2)	29.1 (25.7, 33.6)	29.1 (26.2, 32.1)	29.3 (26.0, 33.2)	29.7 (26.5, 33.6)	30.8 (26.8, 37.5)	31.2 (27.7, 36.9)	30.6 (26.8, 36.2)	**< 0.001**
HEI-2015	52.3 (45.0, 60.6)	54.0 (46.5, 62.0)	53.4 (44.0, 63.2)	53.1 (45.0, 62.0)	53.5 (44.1, 61.1)	54.2 (44.3, 65.2)	55.2 (47.1, 64.4)	54.2 (46.0, 63.9)	0.142
Wear time, h/day	13.1 (12.1, 14.3)	13.8 (12.8, 14.5)	14.7 (13.8, 16.0)	13.8 (12.7, 14.9)	13.0 (11.9, 13.9)	13.4 (12.3, 14.4)	14.4 (13.4, 15.8)	13.5 (12.5, 14.7)	0.121
Sedentary time, h/day	6.3 (5.5, 6.9)	8.1 (7.8, 8.6)	9.9 (9.4, 10.9)	8.0 (6.9, 9.3)	6.8 (6.0, 7.3)	8.5 (8.2, 8.9)	10.2 (9.7, 11.1)	8.6 (7.4, 9.7)	**< 0.001**
LPA, h/day	6.6 (5.6, 7.7)	5.3 (4.4, 6.2)	4.3 (3.3, 5.4)	5.4 (4.3, 6.7)	6.1 (5.0, 7.2)	4.7 (3.8, 5.7)	3.9 (3.1, 5.0)	4.8 (3.7, 6.0)	**< 0.001**
MVPA, min/day	16.7 (7.5, 32.0)	9.0 (3.0, 20.3)	5.8 (1.7, 19.7)	10.3 (3.5, 24.1)	11.5 (5.0, 22.4)	4.6 (2.0, 10.8)	3.2 (1.0, 9.4)	5.7 (1.9, 14.9)	**< 0.001**
HbA1c, %	5.7 (5.4, 5.9)	5.7 (5.4, 5.9)	5.8 (5.4, 5.9)	5.7 (5.4, 5.9)	6.6 (5.9, 7.5)	6.5 (5.9, 7.5)	6.7 (6.1, 7.5)	6.6 (6.0, 7.5)	**< 0.001**
Hypertension	180(41.9)	203(47.5)	220 (50.5)	603 (48.6)	214 (66.4)	232 (65.7)	258 (71.6)	704 (67.9)	**< 0.001**
CVD	44(9.1)	70(13.7)	85 (17.6)	199 (16.0)	73 (19.6)	99 (28.2)	140 (35.4)	312 (30.1)	**< 0.001**
Cancer	32(9.1)	67(14.8)	69 (15.1)	168 (13.5)	40 (14.0)	44 (14.0)	58 (18.2)	142 (13.7)	0.156

Data are n (%) for categoric variables and median (interquartile range) for continuous variables. Percentage and median are adjusted for survey weights of NHANES. Tertiles of sedentary time: participants with prediabetes (h/day): Tertile 1: <7.3; Tertile 2: 7.3–9.0; Tertile 3: ≥ 9.0; participants with diabetes: Tertile 1: <7.7; Tertile 2: 7.7–9.3; Tertile 3: ≥ 9.3. *P values present the difference between prediabetes and diabetes. Boldface type indicates *P <*0.05. *Abbreviate*: *BMI* body mass index, *CVD* cardiovascular disease, *HbA1c* glycated hemoglobin A1c, *HDL-C* high-density lipoprotein cholesterol, *HEI* healthy eating index, *LPA* light-intensity physical activity, *MVPA* moderate- to vigorous-intensity physical activity, *TC* total cholesterol

### Sedentary behavior and all-cause mortality

The participants with prediabetes were followed for a median of 14.1 years (15,781 person-years), with 424 deaths from all-cause and 146 deaths attributable to CVD. Participants with diabetes had a median of 13.5 years of follow-up, accumulating a total of 11,925 person-years with 493 deaths from all-cause and 176 deaths from CVD.

**Fig. 1:**
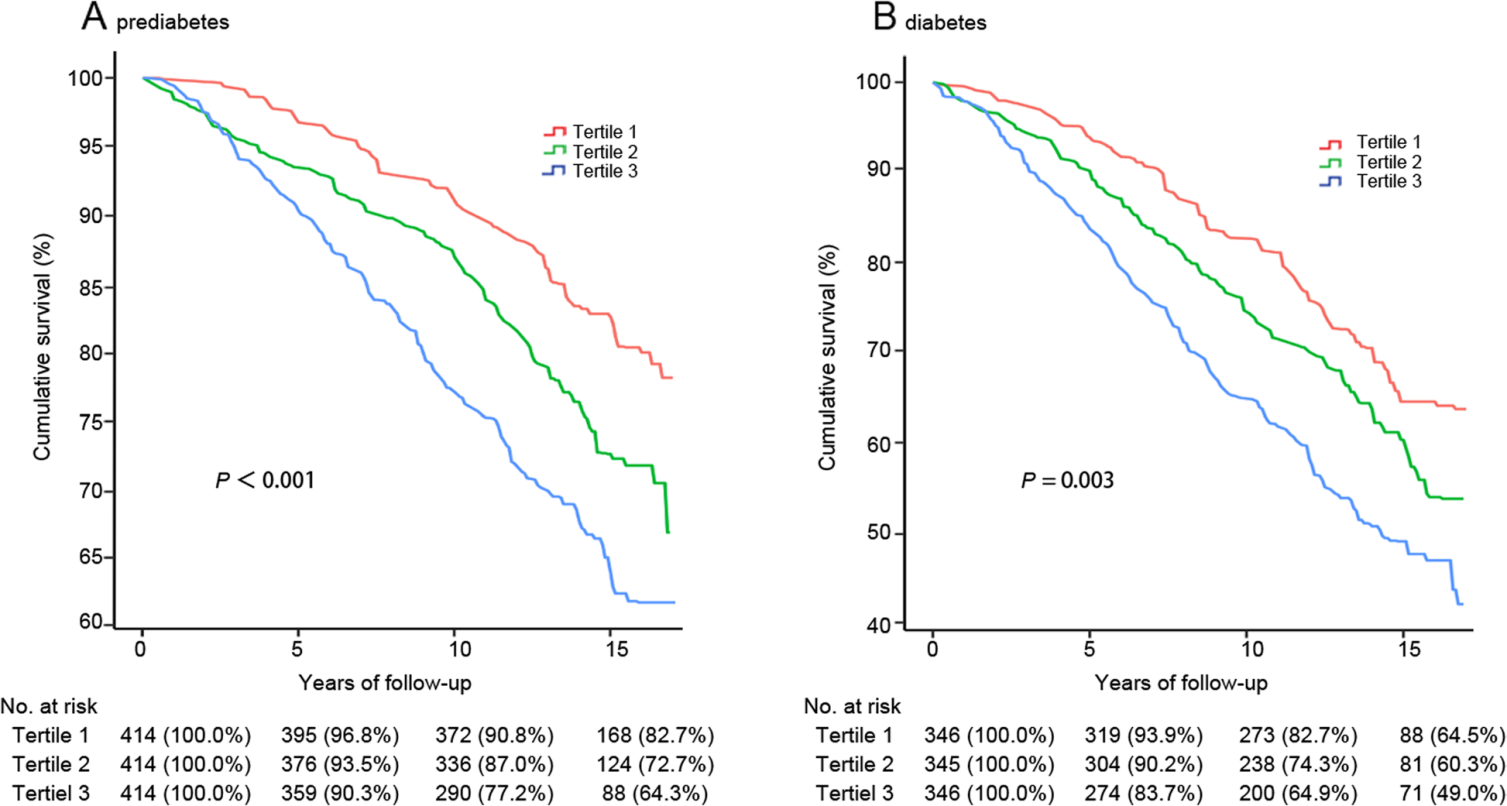
Weighted Kaplan–Meier survival curves for overall mortality according to tertiles of sedentary time among people with prediabetes (**A**) and diabetes (**B**). Tertiles of sedentary time: participants with prediabetes (h/day): Tertile 1: < 7.3, Tertile 2: 7.3–9.0, Tertile 3: ≥ 9.0; Participants with diabetes (h/day): Tertile 1: < 7.7, Tertile 2: 7.7–9.3, Tertile 3: ≥ 9.3. Data are presented as numbers (weighted percentages) for cumulative survival

Among participants with prediabetes, when expressed in tertiles, greater time spent in sedentary behavior was associated with increased all-cause mortality in the partially adjusted model (Table 2; Fig. 1). Adjustment for BMI and MVPA also had little impact on the risk estimate, with participants in the highest tertile of total sedentary time showing a 76% (HR 1.76; 95% CI 1.19, 2.60) higher risk of all-cause mortality than those in the lowest tertile (P for trend = 0.003). We observed parallel results in participants with diabetes, in which the risk increased to 1.24 (95% CI 0.88, 1.77) in the second tertile for sedentary time and additionally increased in the third tertile (HR 1.76; 95% CI 1.17, 2.65) compared with the reference (P for trend = 0.007) after adjusting for age; sex; socioeconomic status; total wear time; BMI; HbA1c level; diabetes duration; glucose-lowering medications; prevalent CVD, hypertension, or cancer; and time spent in MVPA.

**Table 2:** Hazard ratios (95% CI) for all-cause mortality according to tertiles of sedentary time among people with prediabetes and diabetes

Characteristic	Sedentary behavior, h/day			P for trend	Per 60-min increment
	**Tertile 1**	**Tertile 2**	**Tertile 3**		
**Prediabetes (*N* = 1242)**					
Median (IQR)	6.3 (5.5, 6.9)	8.1 (7.8, 8.6)	9.9 (9.4, 10.9)		
No. of deaths/total	87/414	149/414	188/414		
Model 1	1.00 (ref)	1.11 (0.75, 1.64)	2.01 (1.38, 2.94)	0.001	1.25 (1.14, 1.36)
Model 2	1.00 (ref)	1.16 (0.76, 1.76)	1.98 (1.33, 2.93)	0.001	1.24 (1.13, 1.34)
Model 3	1.00 (ref)	1.09 (0.70, 1.69)	1.76 (1.19, 2.60)	0.003	1.19 (1.10, 1.30)
**Diabetes (*N* = 1037)**					
Median (IQR)	6.8 (6.0, 7.3)	8.5 (8.2, 8.9)	10.2 (9.7, 11.1)		
No. of deaths/total	127/346	156/345	210/346		
Model 1	1.00 (ref)	1.24 (0.85, 1.19)	1.84 (1.20, 2.82)	0.004	1.28 (1.16, 1.42)
Model 2	1.00 (ref)	1.31 (0.92, 1.87)	1.80 (1.24, 2.62)	0.002	1.23 (1.12, 1.36)
Model 3	1.00 (ref)	1.24 (0.88, 1.77)	1.76 (1.17, 2.65)	0.007	1.25 (1.12, 1.40)

Model 1: Adjusted for age (10-year intervals), sex (female or male), race/ethnicity (non-Hispanic white, non-Hispanic black, Hispanic Mexican, or others), and total wear time (continuous);

Model 2: model 1 adjustments + potential confounders;

Model 3: model 2 adjustments + BMI (< 25.0, 25.0–29.9, or ≥ 30.0 kg/m^2^) and MVPA (continuous)

Potential confounders include education level (less than high school, high school or equivalent, or college or above), family income-to-poverty ratio (< 1.0, 1.0–3.0, or ≥ 3.0), smoking status (never, former, or current smoker), alcohol consumption (yes or no), HbA1c level (continuous), TC level (continuous), HDL-C level (continuous), prevalence of CVD, hypertension, or cancer (yes or no). Diabetes duration (< 5 or ≥ 5 years) and glucose-lowering medications (insulin, oral antidiabetic agents only, or neither) were specifically adjusted for patients with diabetes

*Abbreviate*: *BMI* body mass index, *CVD* cardiovascular disease, *HbA1c* glycated hemoglobin A1c; *HDL-C* high-density lipoprotein cholesterol, *IQR* interquartile range, *MVPA* moderate- to vigorous-intensity physical activity, *TC* total cholesterol

**Fig. 2:**
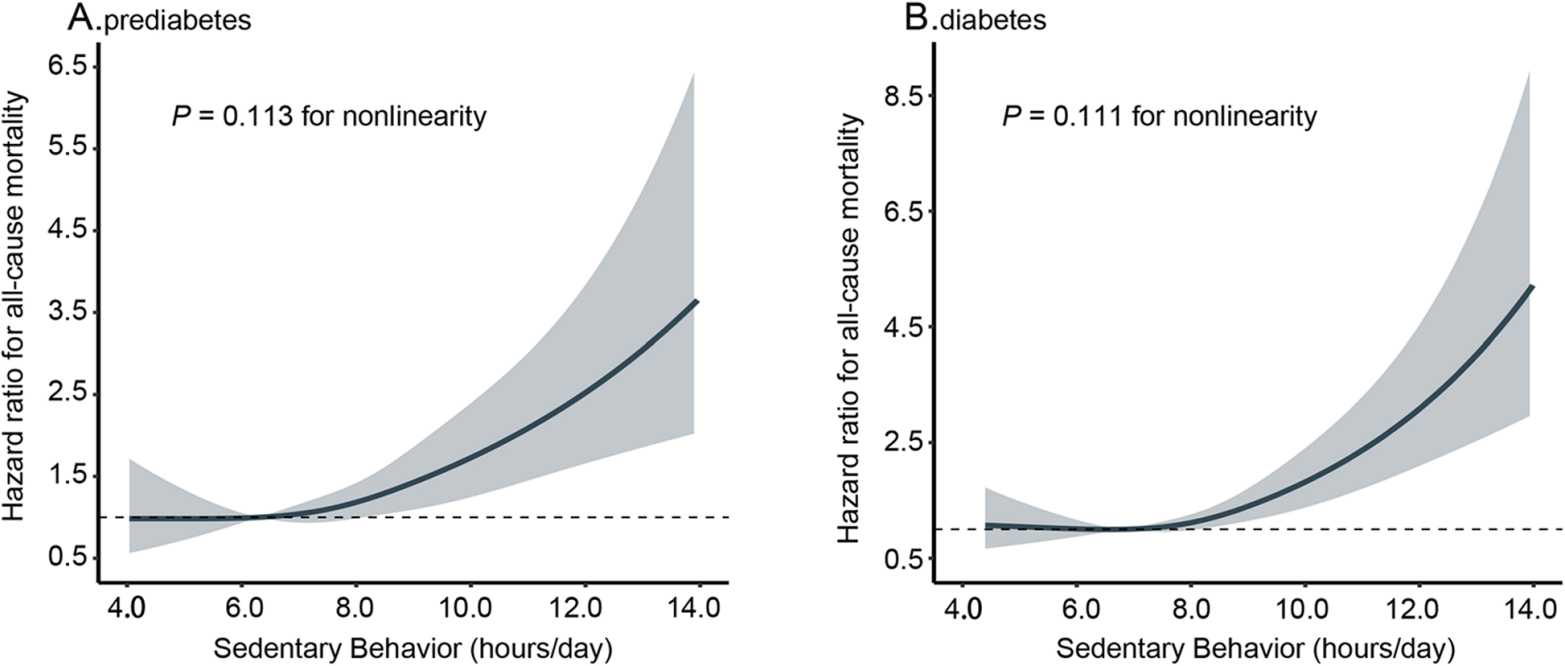
Dose–response associations between sedentary time and all-cause mortality among people with prediabetes (**A**) and diabetes (**B**). A restricted cubic spline regression model with four knots (at the 5th, 35th, 65th, and 95th percentiles) was used to estimate the dose–response association of sedentary time with all-cause mortality. The reference was the median of the lowest tertile of sedentary time. The solid line and gray shading represent hazard ratios and 95% CIs, respectively. Models were adjusted for age, sex, race/ethnicity, education level, family income-to-poverty ratio, smoking status, alcohol consumption, body mass index, glycosylated hemoglobin A1c level, total cholesterol level, high-density lipoprotein cholesterol level, prevalent cardiovascular disease, prevalent hypertension, prevalent cancer, total wear time, and moderate- to vigorous-intensity physical activity. Diabetes duration and glucose-lowering medications were additionally adjusted for patients with diabetes. P-values for nonlinear associations are all ≥ 0.05

We observed a dose–response relationship between sedentary time and the risk of all-cause mortality when sedentary time was expressed continuously (Fig. 2). Among participants with prediabetes, sedentary time was linearly associated with mortality (P-nonlinear = 0.113). Every 60 min/day increment in sedentary time resulted in a 19% higher risk of mortality (HR 1.19; 95% CI 1.10, 1.30). A similar trend toward an association was noted in people with diabetes, with a 25% higher mortality risk for each 60 min/day increase in sedentary time (HR 1.25; 95% CI 1.12, 1.40).

### Subgroup analyses

**Fig. 3:**
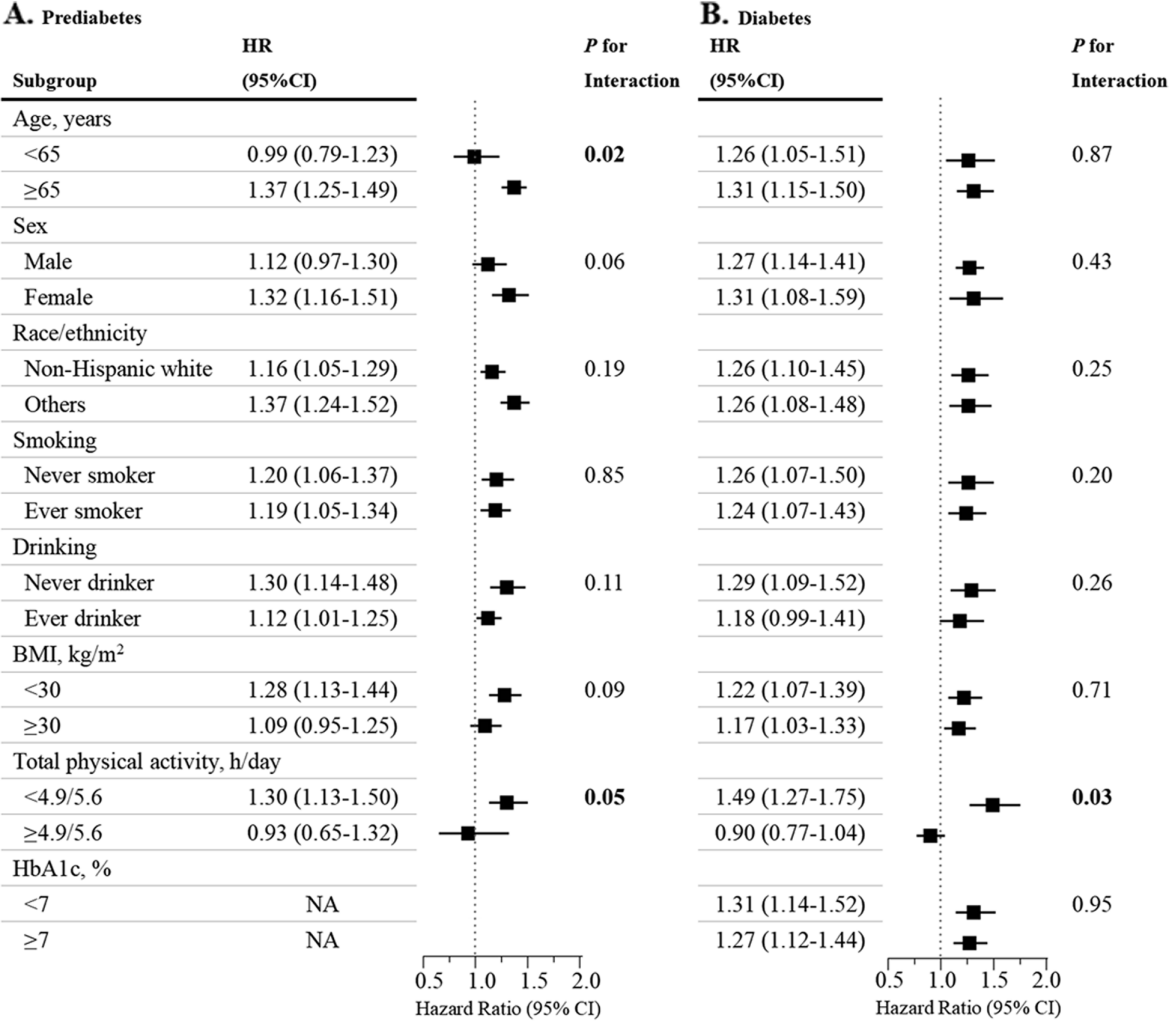
Hazard ratios of all-cause mortality per 60-min increase in sedentary time stratified by potential risk factors among participants with prediabetes (**A**) and diabetes (**B**). Models were adjusted for age, sex, race/ethnicity, education level, family income-to-poverty ratio, smoking status, alcohol consumption, body mass index, glycosylated hemoglobin A1c level, total cholesterol level, high-density lipoprotein cholesterol level, prevalence of cardiovascular disease, prevalence of hypertension, prevalence of cancer, total wear time, and moderate- to vigorous-intensity physical activity except for the corresponding subgroup variables. Low- and high-activity subgroups were determined by sample-weighted medians of total physical activity time (LPA and MVPA). Diabetes duration and glucose-lowering medications were additionally adjusted for patients with diabetes

When the analysis was stratified by total physical activity time, we observed a significant interaction between sedentary time and total physical activity time for all-cause mortality in participants with prediabetes and diabetes (both P for interaction ≤ 0.05) (Fig. 3). The positive association between sedentary behavior and all-cause mortality was more evident among participants with lower activity levels, which were determined based on the sample-weighted median of total LPA and MVPA times. Additional analysis indicated that older adults (age ≥ 65 years) with prediabetes tended to have a higher all-cause mortality risk than younger participants (P for interaction = 0.02). No effect modification by sex, race/ethnicity, smoking status, alcohol consumption, or BMI was identified (all P for interaction > 0.05).

### Isotemporal substitution analyses

**Fig. 4:**
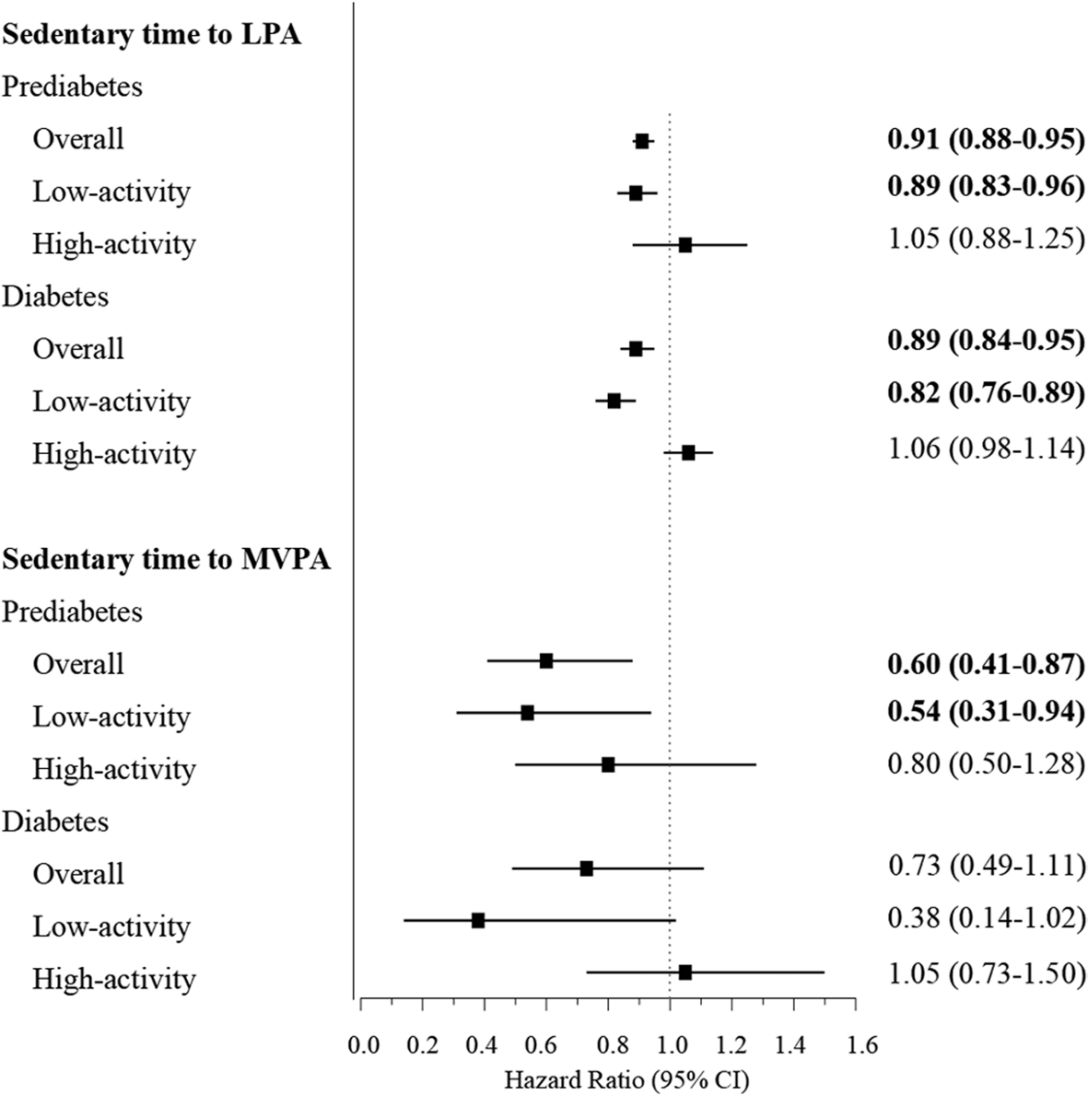
Hazard ratios for all-cause mortality according to isotemporal substitution of 30 min sedentary behavior with equivalent durations of LPA or MVPA among people with prediabetes and diabetes. Low- and high-activity subgroups were determined by sample-weighted medians of total physical activity time (LPA and MVPA). Models omitted the sedentary behavior component and adjusted for age, sex, race/ethnicity, education level, family income-to-poverty ratio, smoking status, alcohol consumption, body mass index, glycosylated hemoglobin A1c level, total cholesterol level, high-density lipoprotein cholesterol level, prevalence of cardiovascular disease, prevalence of hypertension, prevalence of cancer, and total wear time. Diabetes duration and glucose-lowering medications were additionally adjusted for patients with diabetes. Sedentary time was defined as < 100 cpm, LPA as 100–2020 cpm, and MVPA as ≥ 2020 cpm. Boldface type indicates *P* <0.05. cpm, counts per minute; LPA, light-intensity physical activity; MVPA, moderate- to vigorous-intensity physical activity

We investigated the association with mortality risk when statistically replacing 30 min of sedentary behavior with an equivalent time of LPA or MVPA using isotemporal models after adjusting for multiple covariates (Fig. 4). Overall, in adults with prediabetes, each 30-min substitution of reducing sedentary behavior by adding LPA was associated with a HR of 0.91 (95% CI 0.88, 0.95), and each 30-min substitution of sedentary behavior with MVPA resulted in a 40% reduction in all-cause mortality (HR 0.60; 95% CI 0.41, 0.87). Similarly, in participants with diabetes, replacing 30 min of sedentary behavior with an equivalent time of LPA and MVPA was also associated with all-cause mortality risk reduction (HR 0.89; 95% CI 0.84, 0.95 for LPA; HR 0.73; 95% CI 0.49, 1.11 for MVPA). On the contrary, replacing 30 min of MVPA with sedentary behavior was associated with a 67% higher mortality in adults with prediabetes (HR 1.67; 95% CI 1.14, 2.42) and a 36% higher mortality in adults with diabetes (HR 1.36; 95% CI 0.90, 2.05) (Supplementary Table 1).

Additionally, stratified analyses revealed that replacing 30 min of sedentary behavior with an equal amount of time was associated with an 11% risk reduction for LPA replacement (HR 0.89; 95% CI 0.83, 0.96) and a 46% risk reduction for MVPA replacement (HR 0.54; 95% CI 0.31, 0.94) in low-activity adults with prediabetes, while we observed an 18% risk reduction for LPA replacement and a 62% risk reduction for MVPA replacement in those low-activity adults with diabetes (HR 0.82; 95% CI 0.76, 0.89 for LPA, HR 0.38; 95% CI 0.14, 1.02 for MVPA). In contrast, among highly active individuals with prediabetes and diabetes, replacing sedentary behavior with either LPA or MVPA showed no mortality benefit.

### Sensitivity analyses

The positive association between sedentary time and mortality was not significantly altered after excluding deaths within the first 2 years of follow-up or those who had prevalent CVD or cancer (Supplementary Tables 2–4). Similarly, the results remained unchanged when further adjusting for HEI, CRP or mobility limitations (Supplementary Table 5). Consistent findings were observed when samples were combined (Supplementary Table 6). Regarding CVD mortality, we also found that higher amounts of sedentary behavior were significantly associated with an increased mortality risk (Supplementary Table 7).

## Discussion

In this prospective cohort of middle-aged and older individuals with prediabetes and diabetes, higher accelerometer-measured sedentary behavior was significantly associated with an increased risk of all-cause mortality. Theoretically, replacing sedentary time with equivalent amounts of LPA or MVPA was related to lower all-cause mortality risks among individuals with prediabetes. Replacement of sedentary behavior with LPA reduced the risk of total mortality among those with diabetes. Furthermore, the detrimental effects of sedentary behavior tended to be more prominent in participants with lower activity levels.

In the general population, it has been demonstrated that sedentary behavior is associated with all-cause and CVD mortality [8, 33]. A recent harmonized meta-analysis that included nine prospective cohort studies reported that high amounts of sedentary time were associated with higher risks of mortality, especially in individuals with low MVPA levels [7]. Prolonged sedentary behavior could adversely influence metabolic syndrome risk scores and contribute to impaired glucose metabolism, ultimately increasing the risk of incident diabetes [18, 34]. However, evidence regarding the potential health consequences of sedentary behavior is scarce among people with prediabetes and diabetes who are at higher risk of mortality. One prospective cohort study with self-reported data that included 15,645 low-income individuals with diabetes from the Southern Community Cohort Study showed that increased time spent in sedentary behavior was associated with a 21% greater risk of all-cause mortality [16]. However, in the only other prospective study of objectively measured sedentary data and mortality, which included 712 adults with diabetes, 134 deaths were recorded over a median follow-up of 6.6 years, and sedentary time showed no significant association with all-cause mortality after adjusting for diabetes duration or total physical activity [15]. Notably, the weakness of these studies was the self-reported measure of sedentary time or the short period of follow-up, which may have misrepresented the strength of the association between sedentary behavior and health risk [35].

In the present prospective study of a national cohort of people with prediabetes and diabetes, higher accelerometer-based time spent in sedentary behavior was linearly associated with a substantially increased risk of premature mortality. Furthermore, our findings indicated that the association of sedentary time with mortality was modified by the amount of physical activity accumulated and showed a stronger effect of sedentary behavior on all-cause mortality among low-activity adults with prediabetes and diabetes, whereas a high level of activity may mitigate the adverse consequences of sedentary behavior. This discrepancy may be attributed to different distributions of sedentary behavior, LPA, and MVPA between low- and high-activity participants. As was discovered in the general population, there was a significant interaction between sitting and MVPA for mortality risk [36].

Isotemporal substitution analyses consider the finite nature of time and the interrelationships among behaviors to estimate the benefits of reallocating time from one behavior to another [31]. Using these strategies in the general population, previous studies showed that replacing a modest amount of sitting with even LPA was associated with significant decreases in mortality risk among individuals who are less active [22]. The replacement of sedentary behavior with physical activity was recently shown to be associated with improved cardiometabolic health in individuals with prediabetes and diabetes [37–39]. In the Early Activity in Diabetes (ACTID) study, which included 519 adults with newly diagnosed type 2 diabetes, isotemporal substitution analyses showed that the replacement of sedentary behavior with the equivalent amount of LPA or MVPA had beneficial effects on BMI, waist circumference, and HDL-C [37]. In addition, a prospective cohort study including 808 adults at high risk of diabetes from the Walking Away from Type 2 Diabetes trial demonstrated that reallocating time away from sedentary behavior into LPA or MVPA was associated with improved cardiometabolic health, as determined by measurements of 2-h glucose results, TG levels, and clustered cardiometabolic risk scores [38].

Our study is the first to extend these previous findings by demonstrating that replacing 30 min of sedentary time with LPA resulted in a 9% reduction in all-cause mortality, whereas replacing it with MVPA resulted in more substantial mortality reductions in participants with prediabetes. We also showed that among individuals with diabetes, replacing 30 min of sedentary time with LPA reduced all-cause mortality by 11%. Each 30-min substitution of MVPA with sedentary time prominently increased the mortality risk compared to when sedentary time was substituted with MVPA. These findings indicated that the loss of MVPA had a more significant impact on mortality risk when compared to the gain of MVPA from sedentary time. In addition, we found that the health benefits of replacing sedentary behavior with physical activity were stronger among individuals who were less active. Interestingly, we did not observe greater benefits when replacing sedentary behavior with MVPA in adults with diabetes, which has also been identified in patients with chronic kidney disease or heart failure [40, 41]. One possible explanation for the inconsistency in risk reductions of all-cause mortality from LPA and MVPA among participants with diabetes might be partly due to differences in physiological mechanisms, as patients with diabetes are prone to sedentariness and are particularly susceptible to the adverse effects of sedentary behavior because of the high prevalence of cardiometabolic risk factors [42, 43]. It is worth noting that when evaluating confidence intervals in the context of clinically important effect sizes, primary findings provide valuable guidance even if they do not reach statistical significance [44]. The survival benefit of replacing sedentary behavior with MVPA, yet non-significant results, may still have a potential benefit when the confidence interval overlaps with values indicative of a beneficial effect and covers mainly one direction of health outcomes. Prospective studies with larger sample sizes are needed.

Meeting the current recommendations for physical activity (e.g., intensity, types, and volume) remains a critical challenge, particularly among people with diabetes [45]. In this regard, our study offers an additional scientific alternative to improve health outcomes for this patient population, specifically for those who spend a considerable portion of their day in sedentary behavior and may be reluctant or unable to participate in MVPA. It may be feasible to encourage adults with diabetes to replace some sedentary behavior with more practical and accessible forms of physical activity that do not exceed the demands of daily living, such as standing, light gardening, and casual walking, rather than concentrating exclusively on promoting MVPA. In the future, lifestyle interventions targeting the shift from sedentary behavior to LPA among patients with diabetes may be promising research directions [10].

Notable strengths of our study include the population-based sample from a well-characterized US national cohort. Moreover, sedentary behavior, which is generally the default behavior status for older persons with diabetes, was characterized using objective accelerometer data. However, several limitations warrant comment. First, given the observational design, exact conclusions about causality should be interpreted with caution. Second, we only recruited middle-aged and older participants who were at least 40 years of age at baseline, which restricted the generalizability of our findings to younger people with prediabetes and diabetes. Third, the lack of repeated measures of exposure variables might have influenced our results because of changes in behavior between baseline and follow-up. Fourth, Troiano et al. reported that accelerometer-measured physical activity declines across age groups [46]. We chose a unique cut-point to categorize each behavior because of the lack of age-specific cut-points and our relatively small sample size. Fifth, the isotemporal substitution analysis only uses statistical models to estimate mortality benefits for time trade-offs between activities, rather than physically substituting time in actual behavior, which should be confirmed by future well-designed interventional research. Sixth, the sedentary behavior data from the accelerometers worn on the hip in the current study may not capture all activities and is not as accurate as that worn on the thigh. Further investigation using posture-based devices to evaluate sedentary behavior is warranted. Finally, residual confounding may still be present, even after adjusting for a range of important confounding variables.

## Conclusions

In a representative population-based sample of US individuals with prediabetes and diabetes, prolonged sedentary behavior was significantly associated with higher all-cause mortality. Statistically, replacing sedentary behavior with LPA would have a potential health benefit in this high-risk population. These findings highlight the important clinical and public health implications of reducing sedentary time and increasing physical activity tailored for people with prediabetes and diabetes.

## Supplementary Information

**Additional file 1.** 

**Additional file 2.** 

## Data Availability

The data sets that support the findings of the current study are available from the corresponding author upon reasonable request.

## Abbreviations

BMI: Body mass index
CI: Confidence interval
CPM: Counts per minute
CRP: C-reactive protein
CVD: Cardiovascular disease
HbA1c: Glycated hemoglobin A1c
HDL-C: High-density lipoprotein cholesterol
HEI: Healthy eating index
HR: Hazard ratio
LPA: Light-intensity physical activity
MVPA: Moderate- to vigorous-intensity physical activity
NHANES: National Health and Nutrition Examination Survey
ST: Sedentary time
STROBE: Strengthening the reporting of observational studies in epidemiology
TC: Total cholesterol
