# Supplementary figures and images for: Replacing of sedentary behavior with physical activity and the risk of mortality in people with prediabetes and diabetes: a prospective cohort study
Source: Int J Behav Nutr Phys Act. 2023 Jul 6;20:81. doi: 10.1186/s12966-023-01488-0 (PMC10324235; doi:10.1186/s12966-023-01488-0)

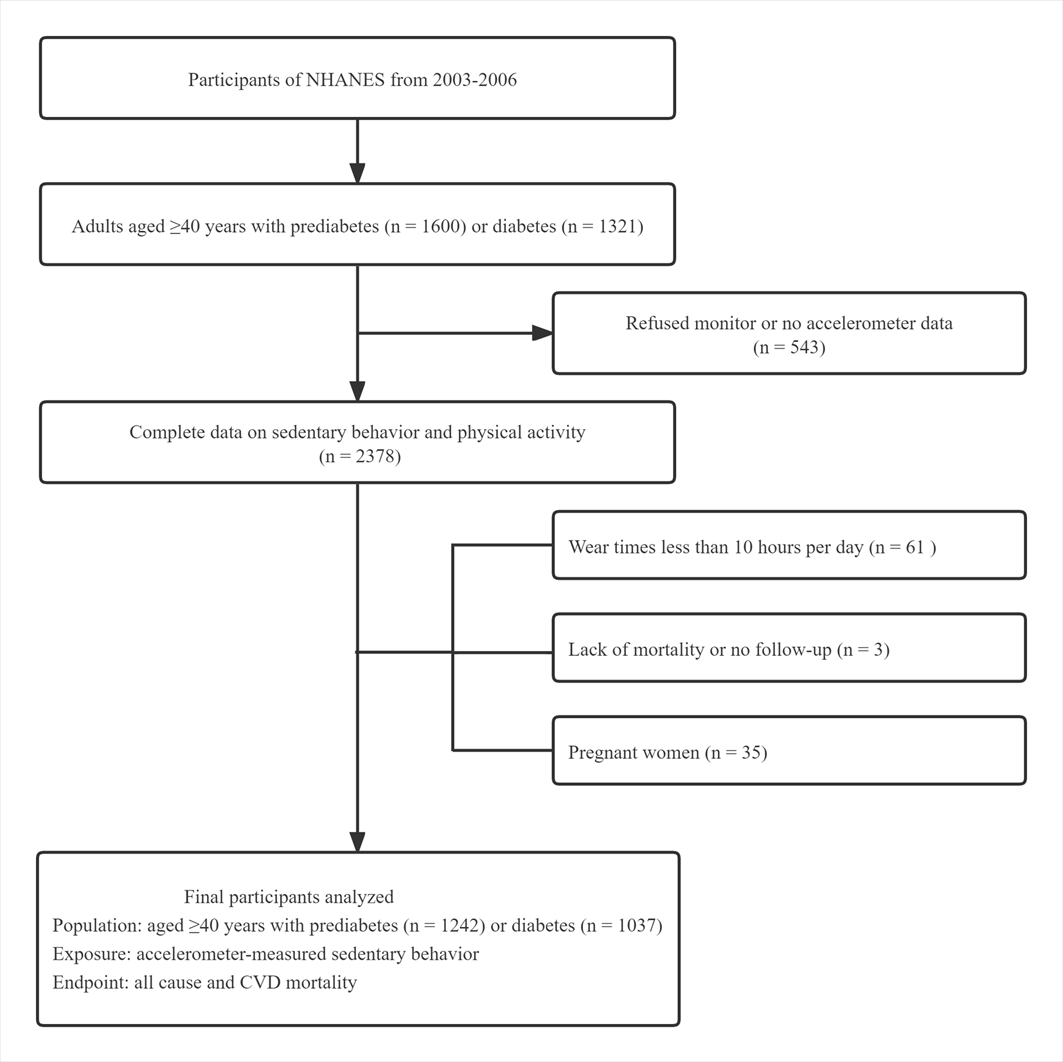

Supplement: Supplementary file 1 — Additional file 1. [file 12966_2023_1488_MOESM1_ESM.tif]
