# Supplementary material for: Replacing of sedentary behavior with physical activity and the risk of mortality in people with prediabetes and diabetes: a prospective cohort study
Source: Int J Behav Nutr Phys Act. 2023 Jul 6;20:81. doi: 10.1186/s12966-023-01488-0 (PMC10324235; doi:10.1186/s12966-023-01488-0)
Supplement: Supplementary file 2 — Additional file 2. [file 12966_2023_1488_MOESM2_ESM.docx]

**Supplementary Material**

**Replacing of sedentary behavior with physical activity and the risk of mortality in people with prediabetes and diabetes: a prospective cohort study**

Ping Zhu^1,3^, MD; Guojuan Lao^2^, MD; Huipeng Li^1^, MS; Rongshao Tan^3^, PhD; Jing Gu^4^, PhD; Jianmin Ran^1,3^, MD

^1^Department of Endocrinology and Metabolism, Guangzhou Red Cross Hospital, Jinan University, Guangzhou, China.

^2^Department of Endocrinology and Metabolism, Sun Yat-sen Memorial Hospital, Sun Yat-sen University, Guangzhou. China.

^3^Institute of Disease-Oriented Nutritional Research, Guangzhou Red Cross Hospital, Jinan University, Guangzhou, China.

^4^Department of Medical Statistics, School of Public Health, Sun Yat-sen University, Guangzhou, China.

**Supplementary Figure 1.** Flow chart.

**Supplementary Table 1.** Hazard ratio (95% CI) for all-cause mortality according to isotemporal substitution of 30 minutes of sedentary time with MVPA or vice versa

**Supplementary Table 2.** Hazard ratio (95% CI) for all-cause mortality according to tertiles of sedentary time among people with diabetes and prediabetes after excluding participants who died with 2 years of follow-up

**Supplementary Table 3.** Hazard ratio (95% CI) for all-cause mortality according to tertiles of sedentary time among people with diabetes and prediabetes after excluding participants with history of CVD

**Supplementary Table 4.** Hazard ratio (95% CI) for all-cause mortality according to tertiles of sedentary time among people with diabetes and prediabetes after excluding participants with history of cancer

**Supplementary Table 5.** Hazard ratio (95% CI) for all-cause mortality according to tertiles of sedentary time among people with diabetes and prediabetes with further adjustment for HEI, mobility limitation, or CRP

**Supplementary Table 6.** Hazard ratio (95% CI) for all-cause and CVD mortality according to tertiles of sedentary time after combining people with diabetes and prediabetes

**Supplementary Table 7.** Hazard ratio (95% CI) for CVD mortality according to quartiles of sedentary time among people with prediabetes and diabetes

Supplementary Table 1. Hazard ratio (95% CI) for mortality according to isotemporal substitution of 30 minutes of sedentary time with MVPA or vice versa

| **Substitution model** | **Hazard ratio**  **(95% CI)** |
| --- | --- |
| **Prediabetes** |  |
| Sedentary time to MVPA | 0.60 (0.41, 0.87) |
| MVPA to sedentary time | 1.67 (1.14, 2.42) |
| **Diabetes** |  |
| Sedentary time to MVPA | 0.73 (0.49, 1.11) |
| MVPA to sedentary time | 1.36 (0.90, 2.05) |

Models were adjusted for age (10-year intervals), sex (female or male), race/ethnicity (non-Hispanic white, non-Hispanic black, Hispanic Mexican, or others), education level (less than high school, high school or equivalent, or college or above), family income-to-poverty ratio (<1.0, 1.0–3.0, or ≥3.0), smoking status (never, former, or current smoker), alcohol consumption (yes or no), BMI (<25.0, 25.0–29.9, or ≥30.0 kg/m2), HbA1c level (continuous), TC level (continuous), HDL-C level (continuous), prevalence of CVD, hypertension, or cancer (yes or no), and total wear time (continuous). Diabetes duration (<5 or ≥5 years) and glucose-lowering medications (insulin, oral antidiabetic agents only, or neither) were specifically adjusted for patients with diabetes. Sedentary time was defined as <100 cpm, LPA as 100–2020 cpm, and MVPA as ≥2020 cpm. cpm, counts per minute; LPA, light-intensity physical activity; MVPA, moderate- to vigorous-intensity physical activity.

Abbreviate: BMI, body mass index; CVD, cardiovascular disease; HbA1c, glycated hemoglobin A1c; HDL-C, high-density lipoprotein cholesterol; MVPA, moderate- to vigorous-intensity physical activity; TC, total cholesterol.

Supplementary Table 2. Hazard ratio (95% CI) for all-cause mortality according to tertiles of sedentary time among people with diabetes and prediabetes after excluding participants who died with 2 years of follow-up

| **Characteristic** | **Sedentary behavior, h/day** | | | **P for trend** | **Per 60-min increment** |
| --- | --- | --- | --- | --- | --- |
|  | **Tertile 1** | **Tertile 2** | **Tertile 3** |  |  |
| **Prediabetes (N = 1213)** |  |  |  |  |  |
| No. of deaths/total | 82/405 | 137/404 | 176/404 |  |  |
| Model 1 | 1.00 (ref) | 1.05 (0.70, 1.57) | 1.97 (1.31, 2.97) | < 0.001 | 1.24 (1.13, 1.37) |
| Model 2 | 1.00 (ref) | 1.11 (0.72, 1.70) | 1.93 (1.25, 2.98) | 0.002 | 1.23 (1.11, 1.35) |
| Model 3 | 1.00 (ref) | 1.03 (0.66, 1.61) | 1.70 (1.12, 2.59) | 0.009 | 1.19 (1.08, 1.30) |
| **Diabetes (N = 995)** |  |  |  |  |  |
| No. of deaths/total | 117/332 | 142/332 | 192/331 |  |  |
| Model 1 | 1.00 (ref) | 1.12 (0.76, 1.66) | 1.70 (1.07, 2.70) | 0.019 | 1.23 (1.11, 1.37) |
| Model 2 | 1.00 (ref) | 1.23 (0.86, 1.77) | 1.73 (1.13, 2.64) | 0.010 | 1.22 (1.10, 1.35) |
| Model 3 | 1.00 (ref) | 1.17 (0.81, 1.68) | 1.67 (1.05, 2.63) | 0.025 | 1.20 (1.06, 1.36) |

Model 1: Adjusted for age (10-year intervals), sex (female or male), race/ethnicity (non-Hispanic white, non-Hispanic black, Hispanic Mexican, or others), and total wear time (continuous);

Model 2: model 1 adjustments + potential confounders;

Model 3: model 2 adjustments + BMI (<25.0, 25.0–29.9, or ≥30.0 kg/m2) and MVPA (continuous).

Potential confounders include education level (less than high school, high school or equivalent, or college or above), family income-to-poverty ratio (<1.0, 1.0–3.0, or ≥3.0), smoking status (never, former, or current smoker), alcohol consumption (yes or no), HbA1c level (continuous), TC level (continuous), HDL-C level (continuous), prevalence of CVD, hypertension, or cancer (yes or no). Diabetes duration (<5 or ≥5 years) and glucose-lowering medications (insulin, oral antidiabetic agents only, or neither) were specifically adjusted for patients with diabetes.

Abbreviate: BMI, body mass index; CVD, cardiovascular disease; HbA1c, glycated hemoglobin A1c; HDL-C, high-density lipoprotein cholesterol; MVPA, moderate- to vigorous-intensity physical activity; TC, total cholesterol.

Supplementary Table 3. Hazard ratio (95% CI) for all-cause mortality according to tertiles of sedentary time among people with diabetes and prediabetes after excluding participants with history of CVD

| **Characteristic** | **Sedentary behavior, h/day** | | | **P for trend** | **Per 60-min increment** |
| --- | --- | --- | --- | --- | --- |
|  | **Tertile 1** | **Tertile 2** | **Tertile 3** |  |  |
| **Prediabetes (N = 1061)** |  |  |  |  |  |
| No. of deaths/total | 68/354 | 107/353 | 133/354 |  |  |
| Model 1 | 1.00 (ref) | 1.07 (0.74, 1.54) | 1.75 (1.14, 2.68) | 0.008 | 1.22 (1.09, 1.36) |
| Model 2 | 1.00 (ref) | 1.15 (0.80, 1.67) | 1.78 (1.09, 2.91) | 0.018 | 1.21 (1.07, 1.36) |
| Model 3 | 1.00 (ref) | 1.07 (0.73, 1.56) | 1.58 (0.98, 2.56) | 0.050 | 1.17 (1.04, 1.30) |
| **Diabetes (N = 765)** |  |  |  |  |  |
| No. of deaths/total | 76/255 | 93/256 | 128/254 |  |  |
| Model 1 | 1.00 (ref) | 0.97 (0.66, 1.45) | 1.71 (0.98, 2.98) | 0.056 | 1.22 (1.07, 1.39) |
| Model 2 | 1.00 (ref) | 0.99 (0.69, 1.44) | 1.93 (1.10, 3.39) | 0.028 | 1.21 (1.06, 1.37) |
| Model 3 | 1.00 (ref) | 0.91 (0.62, 1.33) | 1.77 (1.01, 3.08) | 0.049 | 1.19 (1.03, 1.37) |

Model 1: Adjusted for age (10-year intervals), sex (female or male), race/ethnicity (non-Hispanic white, non-Hispanic black, Hispanic Mexican, or others), and total wear time (continuous);

Model 2: model 1 adjustments + potential confounders;

Model 3: model 2 adjustments + BMI (<25.0, 25.0–29.9, or ≥30.0 kg/m^2^) and MVPA (continuous).

Potential confounders include education level (less than high school, high school or equivalent, or college or above), family income-to-poverty ratio (<1.0, 1.0–3.0, or ≥3.0), smoking status (never, former, or current smoker), alcohol consumption (yes or no), HbA1c level (continuous), TC level (continuous), HDL-C level (continuous), prevalence of hypertension, or cancer (yes or no). Diabetes duration (<5 or ≥5 years) and glucose-lowering medications (insulin, oral antidiabetic agents only, or neither) were specifically adjusted for patients with diabetes.

Abbreviate: BMI, body mass index; CVD, cardiovascular disease; HbA1c, glycated hemoglobin A1c; HDL-C, high-density lipoprotein cholesterol; MVPA, moderate- to vigorous-intensity physical activity; TC, total cholesterol.

Supplementary Table 4. Hazard ratio (95% CI) for all-cause mortality according to tertiles of sedentary time among people with diabetes and prediabetes after excluding participants with history of cancer

| **Characteristic** | **Sedentary behavior, h/day** | | | **P for trend** | **Per 60-min increment** |
| --- | --- | --- | --- | --- | --- |
|  | **Tertile 1** | **Tertile 2** | **Tertile 3** |  |  |
| **Prediabetes (N = 1074)** |  |  |  |  |  |
| No. of deaths/total | 68/358 | 114/359 | 142/357 |  |  |
| Model 1 | 1.00 (ref) | 1.11 (0.73, 1.69) | 1.92 (1.25, 2.94) | 0.003 | 1.21 (1.10, 1.32) |
| Model 2 | 1.00 (ref) | 1.19 (0.76, 1.86) | 1.82 (1.19, 2.78) | 0.005 | 1.18 (1.08, 1.29) |
| Model 3 | 1.00 (ref) | 1.10 (0.68, 1.77) | 1.63 (1.06, 2.51) | 0.022 | 1.15 (1.06, 1.25) |
| **Diabetes (N = 895)** |  |  |  |  |  |
| No. of deaths/total | 103/299 | 124/298 | 174/298 |  |  |
| Model 1 | 1.00 (ref) | 1.12 (0.76, 1.65) | 1.82 (1.16, 2.88) | 0.009 | 1.29 (1.15, 1.43) |
| Model 2 | 1.00 (ref) | 1.16 (0.81, 1.68) | 1.80 (1.22, 2.67) | 0.004 | 1.25 (1.13, 1.38) |
| Model 3 | 1.00 (ref) | 1.05 (0.73, 1.51) | 1.66 (1.13, 2.45) | 0.009 | 1.22 (1.09, 1.37) |

Model 1: Adjusted for age (10-year intervals), sex (female or male), race/ethnicity (non-Hispanic white, non-Hispanic black, Hispanic Mexican, or others), and total wear time (continuous);

Model 2: model 1 adjustments + potential confounders;

Model 3: model 2 adjustments + BMI (<25.0, 25.0–29.9, or ≥30.0 kg/m2) and MVPA (continuous).

Potential confounders include education level (less than high school, high school or equivalent, or college or above), family income-to-poverty ratio (<1.0, 1.0–3.0, or ≥3.0), smoking status (never, former, or current smoker), alcohol consumption (yes or no), HbA1c level (continuous), TC level (continuous), HDL-C level (continuous), prevalence of CVD, or hypertension (yes or no). Diabetes duration (<5 or ≥5 years) and glucose-lowering medications (insulin, oral antidiabetic agents only, or neither) were specifically adjusted for patients with diabetes.

Abbreviate: BMI, body mass index; CVD, cardiovascular disease; HbA1c, glycated hemoglobin A1c; HDL-C, high-density lipoprotein cholesterol; MVPA, moderate- to vigorous-intensity physical activity; TC, total cholesterol.

Supplementary Table 5. Hazard ratio (95% CI) for all-cause mortality according to tertiles of sedentary time among people with diabetes and prediabetes with further adjustment for HEI, mobility limitation, or CRP

| **Characteristic** | **Sedentary behavior, h/day** | | | **P for trend** | **Per 60-min increment** |
| --- | --- | --- | --- | --- | --- |
|  | **Tertile 1** | **Tertile 2** | **Tertile 3** |  |  |
| **Prediabetes** |  |  |  |  |  |
| Participants with data of HEI (N = 1186) | | | | | |
| Model 1 | 1.00 (ref) | 1.09 (0.70, 1.69) | 1.76 (1.19, 2.60) | 0.003 | 1.20 (1.10, 1.30) |
| Model 1 + HEI | 1.00 (ref) | 1.08 (0.69, 1.68) | 1.73 (1.17, 2.56) | 0.003 | 1.19 (1.11, 1.29) |
| Participants with data of mobility limitation (N = 734) | | | | | |
| Model 1 | 1.00 (ref) | 1.13 (0.64, 2.01) | 2.24 (1.28, 3.93) | 0.007 | 1.24 (1.10, 1.39) |
| Model 1 + mobility limitation | 1.00 (ref) | 1.12 (0.63, 1.99) | 2.15 (1.24, 3.73) | 0.010 | 1.23 (1.10, 1.37) |
| Participants with data of CRP (N = 1229) | | | | | |
| Model 1 | 1.00 (ref) | 1.08 (0.69, 1.68) | 1.73 (1.17, 2.56) | 0.004 | 1.19 (1.09, 1.29) |
| Model 1 + CRP | 1.00 (ref) | 1.05 (0.69, 1.60) | 1.67 (1.13, 2.46) | 0.007 | 1.17 (1.08, 1.27) |
| **Diabetes** |  |  |  |  |  |
| Participants with data of HEI (N = 995) | | | | | |
| Model 1 | 1.00 (ref) | 1.26 (0.89, 1.79) | 1.79 (1.20, 2.68) | 0.004 | 1.25 (1.11, 1.40) |
| Model 1 + HEI | 1.00 (ref) | 1.27 (0.90, 1.80) | 1.81 (1.20, 2.74) | 0.005 | 1.25 (1.12, 1.41) |
| Participants with data of mobility limitation (N = 612) | | | | | |
| Model 1 | 1.00 (ref) | 1.00 (0.56, 1.78) | 2.18 (1.33, 3.56) | 0.001 | 1.24 (1.07, 1.42) |
| Model 1 + mobility limitation | 1.00 (ref) | 1.00 (0.55, 1.79) | 2.17 (1.30, 3.62) | 0.002 | 1.24 (1.07, 1.43) |
| Participants with data of CRP (N = 999) | | | | | |
| Model 1 | 1.00 (ref) | 1.20 (0.86, 1.68) | 1.71 (1.14, 2.57) | 0.009 | 1.24 (1.09, 1.39) |
| Model 1 + CRP | 1.00 (ref) | 1.19 (0.84, 1.67) | 1.70 (1.13, 2.56) | 0.010 | 1.23 (1.09, 1.38) |

Model 1: Adjusted for age (10-year intervals), sex (female or male), race/ethnicity (non-Hispanic white, non-Hispanic black, Hispanic Mexican, or others), and total wear time (continuous), education level (less than high school, high school or equivalent, or college or above), family income-to-poverty ratio (<1.0, 1.0–3.0, or ≥3.0), smoking status (never, former, or current smoker), alcohol consumption (yes or no), BMI (<25.0, 25.0–29.9, or ≥30.0 kg/m2), HbA1c level (continuous), TC level (continuous), HDL-C level (continuous), prevalence of CVD, hypertension, or cancer (yes or no), MVPA (continuous). Diabetes duration (<5 or ≥5 years) and glucose-lowering medications (insulin, oral antidiabetic agents only, or neither) were specifically adjusted for patients with diabetes.

Abbreviate: BMI, body mass index; CRP, C-reactive protein; CVD, cardiovascular disease; HbA1c, glycated hemoglobin A1c; HDL-C, high-density lipoprotein cholesterol; HEI, healthy eating index; MVPA, moderate- to vigorous-intensity physical activity; TC, total cholesterol.

Supplementary Table 6. Hazard ratio (95% CI) for all-cause and CVD mortality according to tertiles of sedentary time after combining people with diabetes and prediabetes

| **Characteristic** | **Sedentary behavior, h/day** | | | **P for trend** | **Per 60-minute increment** |
| --- | --- | --- | --- | --- | --- |
|  | **Tertile 1** | **Tertile 2** | **Tertile 3** |  |  |
| **All-cause mortality** |  |  |  |  |  |
| No. of deaths/total | 208/760 | 304/759 | 405/760 |  |  |
| Model 1 | 1.00 (ref) | 1.20 (0.93, 1.53) | 2.15 (1.61, 2.86) | ＜0.001 | 1.28 (1.21, 1.36) |
| Model 2 | 1.00 (ref) | 1.24 (0.97, 1.58) | 2.02 (1.53, 2.68) | ＜0.001 | 1.24 (1.18, 1.30) |
| Model 3 | 1.00 (ref) | 1.14 (0.91, 1.44) | 1.82 (1.39, 2.39) | ＜0.001 | 1.22(1.16, 1.28) |
| **CVD mortality** |  |  |  |  |  |
| No. of deaths/total | 66/563 | 97/560 | 159/561 |  |  |
| Model 1 | 1.00 (ref) | 1.42 (0.98, 2.06) | 2.60 (1.73, 3.90) | ＜0.001 | 1.35 (1.22, 1.50) |
| Model 2 | 1.00 (ref) | 1.36 (0.90, 2.05) | 2.36 (1.52, 3.66) | ＜0.001 | 1.28 (1.17, 1.41) |
| Model 3 | 1.00 (ref) | 1.26 (0.86, 1.84) | 2.02 (1.34, 3.06) | ＜0.001 | 1.23 (1.13, 1.35) |

Model 1: Adjusted for age (10-year intervals), sex (female or male), race/ethnicity (non-Hispanic white, non-Hispanic black, Hispanic Mexican, or others), and total wear time (continuous);

Model 2: model 1 adjustments + potential confounders;

Model 3: model 2 adjustments + BMI (<25.0, 25.0–29.9, or ≥30.0 kg/m^2^) and MVPA (continuous).

Potential confounders include education level (less than high school, high school or equivalent, or college or above), family income-to-poverty ratio (<1.0, 1.0–3.0, or ≥3.0), smoking status (never, former, or current smoker), alcohol consumption (yes or no), HbA1c level (continuous), TC level (continuous), HDL-C level (continuous), prevalence of CVD, hypertension, or cancer (yes or no).

Abbreviate: BMI, body mass index; CVD, cardiovascular disease; HbA1c, glycated hemoglobin A1c; HDL-C, high-density lipoprotein cholesterol; MVPA, moderate- to vigorous-intensity physical activity; TC, total cholesterol.

Supplementary Table 7. Hazard ratio (95% CI) for CVD mortality according to quartiles of sedentary time among people with prediabetes and diabetes

| **Characteristic** | **Sedentary behavior, h/day** | | | |
| --- | --- | --- | --- | --- |
|  | **Quartile 1** | **Quartile 2** | **Quartile 3** | **Quartile 4** |
| **Prediabetes (N = 964)** |  |  |  |  |
| No. of deaths/total | 21/241 | 26/241 | 44/241 | 55/241 |
| Model 1 | 1.39 (0.82, 2.35) | 1.00 (ref) | 1.42 (0.84, 2.40) | 2.87 (1.64, 5.01) |
| Model 2 | 1.21 (0.64, 2.27) | 1.00 (ref) | 1.34 (0.76, 2.37) | 2.63 (1.51, 4.57) |
| Model 3 | 1.31 (0.68, 2.52) | 1.00 (ref) | 1.15 (0.63, 2.11) | 2.02 (1.14, 3.58) |
| **Diabetes (N = 720)** |  |  |  |  |
| No. of deaths/total | 25/180 | 40/180 | 49/180 | 62/180 |
| Model 1 | 1.00 (ref) | 1.52 (0.80, 2.91) | 2.85 (1.24, 6.54) | 3.18 (1.59, 6.39) |
| Model 2 | 1.00 (ref) | 1.41 (0.72, 2.75) | 2.31 (0.97, 5.51) | 2.40 (1.07, 5.37) |
| Model 3 | 1.00 (ref) | 1.36 (0.72, 2.58) | 2.29 (1.03, 5.05) | 2.45 (1.07, 5.63) |

Model 1: Adjusted for age (10-year intervals), sex (female or male), race/ethnicity (non-Hispanic white, non-Hispanic black, Hispanic Mexican, or others), and total wear time (continuous);

Model 2: model 1 adjustments + potential confounders;

Model 3: model 2 adjustments + BMI (<25.0, 25.0–29.9, or ≥30.0 kg/m2) and MVPA (continuous).

Potential confounders include education level (less than high school, high school or equivalent, or college or above), family income-to-poverty ratio (<1.0, 1.0–3.0, or ≥3.0), smoking status (never, former, or current smoker), alcohol consumption (yes or no), HbA1c level (continuous), TC level (continuous), HDL-C level (continuous), prevalence of CVD, hypertension, or cancer (yes or no). Diabetes duration (<5 or ≥5 years) and glucose-lowering medications (insulin, oral antidiabetic agents only, or neither) were specifically adjusted for patients with diabetes.

Abbreviate: BMI, body mass index; CVD, cardiovascular disease; HbA1c, glycated hemoglobin A1c; HDL-C, high-density lipoprotein cholesterol; MVPA, moderate- to vigorous-intensity physical activity; TC, total cholesterol.
